# Knowledge Valuation by Iranian Women with High-Risk Pregnancy: A Qualitative Content Analysis

**DOI:** 10.30476/IJCBNM.2020.83305.1139

**Published:** 2020-07

**Authors:** Zahra Shojaeian, Talat Khadivzadeh, Ali Sahebi, Hossein Kareshki, Fatemeh Tara

**Affiliations:** 1 Department of Midwifery, School of Nursing and Midwifery, Mashhad University of Medical Sciences, Mashhad, Iran; 2 Nursing and Midwifery Care Research Center, Mashhad University of Medical Sciences, Mashhad, Iran; 3 Assistant Professor of Clinical Psychology, Senior Faculty of The William Glaser institute, Sydney, Australia; 4 Department of Counseling Educational Psychology, School of Educational Sciences and Psychology, Ferdowsi University of Mashhad, Mashhad, Iran; 5 Research Center for Patient Safety, Mashhad University of Medical Sciences, Mashhad, Iran

**Keywords:** High-risk pregnancy, Knowledge, Valuation

## Abstract

**Background:**

Gaining adequate knowledge about high-risk pregnancy (HRP) and correct understanding of the condition, empowers women to improve the use of prenatal care, practice better self-care, and reduce the risk of pregnancy complications. The present study aimed to assess the valuation of knowledge by Iranian women with HRP

**Methods:**

The present qualitative study was conducted on 25 women with HRP from August 2017 to August 2018 at various educational hospitals in Mashhad, Iran. The data were collected through in-depth semi-structured interviews. The data collection process continued until data saturation. The data were analyzed using the conventional content analysis method and MAXQDA software (version10.0). Data collection and data analysis were conducted concurrently

**Results:**

Based on the analysis of the interviews, two main categories and eight sub-categories were extracted. The main categories were “Positive valuation of credible problem-relevant knowledge” and “Avoidance of misleading and stressful information”. The results showed that various factors had a positive impact on knowledge gathering, namely personal and other people’s experiences, obtaining need-based information, sympathetic advice from others, and faith-based health recommendations. On the other hand, factors that had a negative impact were related to the type of information that caused stress, was unreliable, inefficient or incompatible with personal goals.

**Conclusion:**

The findings of the present study would help health care providers to offer suitable and empathetic counseling to women with HRP. Providing valid and accessible sources of information will lead to faster and timely referrals of such patients.

## INTRODUCTION

Pregnancy is defined as high-risk if the mother has been pregnant more than four times, is aged under 18 or above 35 years, or has at least one medical condition before/during pregnancy. These risk factors are directly associated with increased maternal and fetal mortality and morbidity. ^1^ According to the World Health Organization (WHO), 830 women die daily due to pregnancy or childbirth complications. ^2^ The prevalence of high-risk pregnancy (HRP) varies from country to country. The reported maternal death due to at least one risk factor in the Iranian provinces of Kermanshah and Hormozgan was 39.8% and 74.7%, respectively. ^3 , 4^ Besides maternal death, fetal complications (e.g., fetal death) due to HRP may also increase. ^1^ Despite the obvious need of such women for better care and awareness of the risks, some women nonetheless consciously opted to get pregnant. Somehow, they were still motivated to do so and resorted to various strategies to increase the chances of getting pregnant and hoped for a healthy baby.

The precautions pregnant women take to safeguard both their own and their fetuses health does not necessarily mean that these are in line with medical recommendations. ^5^ Various studies have assessed the impact of maternal knowledge on pregnancy complications and the associated risk factors. ^6 - 8^ Maternal knowledge and fertility behavior is influenced by various factors such as culture and religion, ^9^ values and preferences, ^10^ the source of information, and the behavior of health care providers. ^6^ It has been shown that improved interaction with caregivers is achieved if they better understand the mindset of pregnant women and those factors affecting unhealthy behaviors during pregnancy. ^11^ Various studies in Iran have assessed different sources of information and the level of maternal knowledge among pregnant women. ^12 , 13^ However, none of these studies have addressed the valuation of knowledge by women with HRP. Gaining adequate knowledge about HRP and correct understanding of the condition, empowers women to improve the use of prenatal care, practice better self-care, and reduce the risk of pregnancy complications.

Given that qualitative approaches provide a rich and deep description of the participants’ experiences and their context, the qualitative content analysis method was used to comprehend and determine the factors affecting knowledge valuation. The objective of the present qualitative study was to explore the valuation of knowledge by Iranian women with HRP. We trust that the outcome of the study will increase awareness of health care providers and improves self-care skills through a pregnancy care program.

## PATIENTS AND METHODS

The present qualitative study, using the content analysis method, was conducted from August 2017 to August 2018 in Mashhad, Iran. The target population was women referred to educational hospitals and health centers in Mashhad. The purposive sampling method was used to recruit the participants and the sampling was continued until data saturation. The inclusion criteria were Iranian mothers with HRP and women who were at risk for high-risk pregnancy and trying to get pregnant, ability to understand and speak Persian, willingness to share their experience, and capable of verbal communication to provide complete information. The exclusion criteria were unwillingness to participate in the study and any congenital fetal abnormalities. Based on these criteria, a total of 25 women with HRP were recruited into the study. 

*Data Collection:* The data were collected through 29 individual in-depth semi-structured interviews. The face-to-face interviews were held in a private and calm setting at our center and each lasted between 25 to 50 minutes. The interviews were conducted by the first author following approval from the study supervisor. The data collection process continued until data saturation, i.e., no new substantive code or new category was acquired. Note that since the present study was a subset of the main research program, only part of the collected data is presented herein. 

The interview started with general questions such as “What did you know about pregnancy before getting pregnant?”, “What was your perception of pregnancy?”, and “What are your positive and negative pregnancy experiences?” The interview was then continued with targeted questions, “What should you have known about pregnancy?”, “Which pregnancy-related recommendations did you consider useful, and why?”, “Which recommendations were acceptable or unacceptable to you, and why?”, “What is the most important reason for you to adhere to medical recommendations?”, “What is your perception of the provided medical recommendations in high-risk pregnancy?”, and “Do you consider a pregnancy education program useful?”. With the permission of the participants, an audio recording of the interviews was made. Note that after interviewing all the 25 participants, an additional interview was conducted with 4 of the 25 participants for further clarification. Therefore, the data were collected through 29 in-depth semi-structured interviews. 

*Data Analysis:* The data were analyzed using the conventional content analysis method as described by Elo and Kingas (2008). ^14^ The analysis involved three phases, namely preparation, organization, and reporting. In the preparation phase, each interview was transcribed verbatim, followed by a repeated study of the text to gain in-depth understanding (immersion) of the data. The organization stage included the selection of semantic units and codes, grouping related codes, categorization of similar groups, and finally the abstraction process to form main categories. The final stage involved reporting the extracted categories. This process was a joint effort by all the research team members and the final list of categories was formulated after internal discussions and multiple joint meetings.

*Rigor:* Data trustworthiness was assessed using the four criteria proposed by Lincoln and Guba, namely credibility, dependability, transferability, and confirmability. ^15^ The credibility criterion was achieved by selecting participants with maximum variation in terms of risk factors as well as by re-evaluation of the extracted codes together with the participants to ensure accuracy. To ensure dependability, the data were continuously evaluated. To improve the level of transferability, information such as demographic characteristics, study conditions, interview techniques, data collection methods, accurate quoting of participants’ statements, researcher’s observations, and the data analysis process were adequately described. To ensure conformability, all steps involved in data collection and data analysis were continuously recorded in support of an audit trail. The MaxQDA software (version 10.0) was used for data management.

The present study was approved by the Ethics Committee of Mashhad University of Medical Sciences, Mashhad, Iran (code: IR.MUMS.REC.1396.126). The participants were informed about the goals and methodology of the research. The confidentiality of any disclosed information was guaranteed. Participants were permitted to withdraw from the study for any reason. Written informed consent was obtained from all the participants.

## RESULTS

A total of 25 mothers with HRP and women who were at risk for high-risk pregnancy, aged 17 to 41 years, participated in the study. The demographic characteristics of the participants are listed in Table 1.

**Table 1:** Demographic characteristics of the participants (N=25)

Participant	Age (years)	Qualification	Occupation	Gravidity (number)	Pregnancy risk factor
1	41	BSc	Midwife	3	>35 years
2	37	MSc	Midwife	3	>35 years, Incompetent cervix
3	38	BSc	Self-employed	3	>35 years
4	28	High school	Housewife	2	Diabetes
5	29	BSc	Self-employed	1	Heart disease
6	37	PhD	Accountant	1	>35 years, Multiple sclerosis
7	32	MSc	Teacher	2	Heart disease, Recurrent abortion
8	24	High school diploma	Housewife	4	Recurrent abortion
9	17	High school diploma	Housewife	0	>18 years, Heart disease,
10	29	Diploma	Housewife	1	Heart disease
11	34	Diploma	Housewife	2	Asthma
12	17	High school diploma	Housewife	0	>18 years, Heart disease,
13	17	Primary school certificate	Housewife	0	>18 years
14	39	Primary school certificate	Housewife	5	>35 years, Recurrent Stillbirth
15	40	Primary school certificate	Housewife	3	>35 years
16	24	Primary school certificate	Housewife	5	Recurrent abortion
17	39	Primary school certificate	Housewife	1	>35 years, History of depression
18	37	BSc	Housewife	6	>35 years, Recurrent abortion
19	28	Diploma	Self-employed	4	Recurrent abortion
20	38	Primary school certificate	Housewife	4	>35 years, Recurrent abortion, History of uterine surgery
21	21	Diploma	Housewife	3	Stillbirth
22	17	High school diploma	Housewife	1	<18 years
23	38	BSc	Housewife	1	>35 years
24	37	Diploma	Self-employed	4	>35 years, Cesarean section>3
25	26	Diploma	Self-employed	0	Recurrent abortion

Analysis of the 29 interviews resulted in two main categories and eight sub-categories. The two main categories were “Positive valuation of credible problem-relevant knowledge” and “Avoidance of misleading and stressful information” (Table 2). 

**Table 2:** Main categories and sub-categories extracted from the interview data

Sub-categories	Main categories
Obtaining relevant information on a need basis	Positive valuation of credible problem-relevant knowledge
Decision-making based on personal and other people’s experiences	
Positive approach to sympathetic advice	
Trust in faith-based health advice	
Avoiding stressful information	Avoidance of misleading and stressful information
Refusing advice contradictory to personal knowledge	
Ignoring advice incompatible with personal goals	
Distrust in public health care recommendations	

### *1. Positive Valuation of Credible Problem-relevant Knowledge*

#### *1.a. Obtaining Relevant Information on a Need Basis*

Most of the participants evaluated the received information based on their pregnancy needs and subsequently classified them as valid or invalid. The urge to obtain certain information was in line with their current problems. Therefore, they selectively searched for the required information from all available sources and knowingly chose the one that directly met their needs. A participant stated:

*“It is customary that physicians provide pregnant women with a standard list of recommendations. During a visit, I only ask questions that are important to me and only pay attention to those recommendations relevant to my questions.”* (P4)

#### *1.b. Decision-making Based on Personal and other People’s Experiences *

The participants adopted the strategy to rate the received information based on its source. Information received from mothers, as well as the knowledge gained from their previous pregnancies, were rated positively. Two participants stated:

*“Based on my personal experience, I do recommend that pregnant women with a history of abortion to monthly undergo sonography up to the 30th week of pregnancy. I also believe that they should lay down as much as possible.”* (P2)

*“I accept the advice from other pregnant women who, like me, suffer from asthma. I will seek advice from a family member who was in the same situation.”* (P11)

Most of the participants had more trust in the advice given by experienced physicians, particularly those treated many patients. A participant stated:

*“I am very much influenced by my physician’s advice. She has treated many pregnant women, conducts a thorough examination, and assesses my medical test reports.”* (P3)

#### *1.c. Positive Approach to Sympathetic Advice*

Most of the participants tended to learn from the experience of others and were influenced by the information provided. They were positive about and listened to the advice given since they believed it to be genuine and supportive.

*“In a sympathetic tone, my mother asked me not to go to work as it may endanger my pregnancy. Like a typical mum, she advised me to stay home and rest for 4 months.”* (P1)

#### *1.d. Trust in Faith-based Health Advice*

Of the participants who had access to the internet, some searched various web sites to find answers to their pregnancy-related questions. Interestingly, they tended to accept recommendations from religious websites, but at the same time required authentication and confirmation of the information from other sources. 

*“I mainly search and trust religious websites to find answers to my pregnancy-related questions, e.g., which verses of the Qur’an should I read during the first or second months of pregnancy. In case of additional questions, I ask other people and then decide on what to do next.”* (P16)

Overall, the first main category indicated that the positive valuation of knowledge by women with HRP was based on a positive response to recommendations, personal and other people’s experiences, availability of the relevant information, religious teachings, and needs during pregnancy.

### *2. Avoidance of Misleading and Stressful Information*

#### *2.a. Avoiding Stressful Information*

Most participants refrained from asking pregnancy-related questions to others since their behavior and manner of providing information created stress. In general, they ignored any information that caused them stress. A participant stated:

*“I avoid getting information from my in-laws as they make me too tense with their responses. I made this mistake during my previous pregnancy that resulted in a miscarriage. This time, I simply ignore them.”* (P20)

Some participants would even avoid visiting health centers if the information given by the personnel was perceived as stressful. This meant that their questions would remain unanswered and necessary medical care had to be postponed. A participant stated:

*“Last time I visited a health center for my pregnancy check-up, my maternity record was labeled *“high-risk pregnancy”*. This caused me so much stress to the extent that I avoided further visits to the center and to my physician. Even if the information is correct, sometimes it is better not to know the truth.”* (P5)

#### *2.b. Refusing Advice Contradictory to Personal Knowledge *

Some participants viewed the knowledge obtained from self-selected sources of information as trustworthy and even scientific, regardless of the source and credibility of the information. Consequently, anything contradictory to their knowledge was rejected and considered unscientific. One of the participants stated:

*“My physician claims that pregnancy worsens diabetes, but I know of other diabetic women that their disease had improved after getting pregnant. I am a logical person, but in this case, the physician could be wrong. I could be among those with improved diabetes after pregnancy.”* (P4)

#### *2.c. Ignoring Advice Incompatible with Personal Goals*

Having a child was the main goal in life for all our participants and they planned it in detail. The strive for pregnancy was to such an extent that any recommendations made by the medical staff incompatible with their goals were immediately rejected. At times, they trusted in the supernatural power of God and continued their pursuit of pregnancy. A participant stated: 

*“Despite the advice of my physician, I did my utmost to get pregnant and had full trust in God to succeed. I believe God does have the power to heal our physical problems.”* (P14) 

Some other participants had set themselves the personal goal of getting pregnant despite specific discouragement from physicians and treatment teams. This was to the extent that it could endanger their lives, or even result in death. A participant stated: 

*“My husband is a kind-hearted and selfless man. He has done a lot for me and in return I wanted him to experience fatherhood. This is not out of pity for him. It is my duty to have a child and he has the right to be a father. After giving birth to our first child, my physician specifically requested not to get pregnant again. But I did and I was even willing to opt for surrogacy in case of problems.”* (P19)

#### *2.d.Distrust in Public Health Care Recommendations *

Some of the participants opted for the private health sector instead of public health centers despite the extra costs. They considered the services provided in public health centers were inadequate in terms of specialized medical personnel and equipment and the lack of respect for human dignity. A participant stated:

*“In the public health system, physicians tend to be arrogant, ignore the patients, and limit prescribing medications and medical tests. As soon as they learned that I am classified as a high-risk pregnancy case, they immediately referred me to another physician and consequently, I was forced to go through the whole admission process again. I tend not to discuss my medical problems anymore as I need to repeat myself to an array of people.”* (P6)

During the interviews, we noticed that some participants held back certain type of information for multiple reasons. Some preferred not to share the information, could not remember, or considered them as unimportant. 

## DISCUSSION

The present study aimed to assess the valuation of knowledge by women with HRP. The results showed that various factors had a positive impact on knowledge gathering, namely personal and other people’s experiences, obtaining need-based information, genuine advice from others, and faith-based health recommendations. On the other hand, factors that had a negative impact were related to the type of information that caused stress, was discouraging, or incompatible with personal goals. 

The participants generally valued the advice from physicians and treatment teams, however, they preferred to receive the advice from physicians with extensive practical experience. A study conducted in Saudi Arabia indicated that two-thirds of women with HRP acknowledged having trust in the advice and information given by physicians. ^6^ Similarly, another study reported that 71.6% of women received the required information from physicians and more than one-third believed the provided information was helpful. ^8^ In line with our results, a previous study reported that the advice given by health care providers was among the nine factors influencing the attitude of women with a pregnancy risk factor (e.g., advanced age). ^16^

Family and relatives were stated as the main source of information by our participants. Similarly, a study conducted in Canada among female immigrants reported that they considered family members and health care providers as the primary source of nutritional and behavioral information. ^17^ Another study reported that the source of information and motivation for adhering to pregnancy and delivery recommendations mainly came from physicians followed by family members, friends, and literature. ^18^ The use of herbal medicine during pregnancy was typically promoted by friends and family members which formed the best source of information and an important incentive for pregnant women. ^19^ In line with our findings, it has been reported that the information provided through social media played a significant role in fertility, particularly the first childbirth. ^20^

Generally, faith-based recommendations were more accepted by women with HRP. It is a fact that faith plays a significant role in facilitating or hinder the acceptance of recommendations. It has been reported that religious teachings and faith during pregnancy positively affected the attitude of women toward pregnancy. ^21 , 22^ In a study conducted in Canada on the role of various religions in decision-making, religious beliefs among Muslims and recognition of religious diversity and cultural functions were emphasized. ^9^

Our results showed that the participants were very selective in the admittance of information and only accepted advice that met their needs at the time. In Switzerland, epileptic women gathered relevant information about the required medicine and selected a drug they deemed appropriate. In line with our findings, inconsistency between the obtained information (e.g., drug use) hindered their decision-making process. ^23^ We also found that knowledge gained through valid (or invalid) observations was another source of decision-making. A previous study showed that pregnant women who were unaware of the importance of their body mass index refrained from losing weight or declined the required intervention despite their physician’s advice. ^7^ Another study found that women aged 35 years and over, despite having more experience and knowledge, did not have a better understanding of pregnancy risks compared to younger women. ^24^ In the above-mentioned studies, the lack of information was the main reason for refusing advice. 

Women with HRP refused any advice incompatible with their personal goals in life. For some, the fulfillment of their marital duties was paramount to the extent of wanting to get pregnant even if it would endanger their lives. ^7^ In line with our findings, a previous study reported that despite being given detailed information and advice about epilepsy, female patients did not refrain from getting pregnant. ^25^ Another study showed that fertility behaviors in many societies are largely influenced by fertility preferences. ^26^ Seemingly, childbearing preference in women with HRP is directly related to fertility behaviors in various societies.

In line with our findings, avoidance was one of the stress coping strategies during pregnancy. ^27^ Seeking information to reduce stress levels in problematic situations helps when people encounter controllable stressors. ^28^ Therefore, the reaction of our participants could be due to uncontrollable stress caused by pregnancy risks. In contrast with our findings, a study concluded that the provision of information about pregnancy risks and the use of screening methods, on the one hand, increased the feeling of duality and, on the other hand, the feeling of calmness in women. ^29^ The reaction of each person to stressors is associated with genetic factors, personality traits (e.g., assessment of the stressor, coping strategies), living conditions and environment, social support structures, and past experiences. ^30^ Per individual, the difference in response was related to variation in any of the above-mentioned parameters.

Some participants did not believe in the effectiveness of the advice given by health care providers, which hampered the transfer of information. In line with our findings, a study in Ghana on women’s perception of the health care system reported shortcomings in terms of unfriendly behavior of caregivers, incompatibility of the provided service with the local culture, long waiting time, limited childbirth delivery options, and disrespect for patient privacy. ^31^ For pregnant women, physicians and medical personnel are usually the best sources of valid information. ^6 , 8^ Wilhelm and colleagues concluded that the behavior of caregivers toward pregnant women should be proportional to their emotional status, personal wishes, and in tune with their personal and family experiences. ^32^ Pregnant women are generally in need of an empathic interaction, openness to discuss various issues, and adequate time from caregivers. ^33^ Health education compatible with patient’s needs is reported as an effective method. ^34^

As the main limitation, due to the nature of qualitative studies, our findings cannot be generalized. The main strength of the present study lay in assessing knowledge valuation in high-risk pregnancies. However, this strength is undermined by the generalizability issue inherent to qualitative studies.

## CONCLUSION

The factors that had a positive impact on knowledge valuation by women with HRP were need-based information, sympathetic recommendations by others, and faith-based health advice. On the other hand, some factors had a negative impact such as stress-causing information, information considered discourteous, and incompatible with personal goals. Understanding the above would help health care providers to offer suitable and empathetic counseling. Providing valid and accessible sources of information will lead to faster and timely referrals of women with HRP.
